# Supplementary material for: The Memristive Properties and Spike Timing-Dependent Plasticity in Electrodeposited Copper Tungstates and Molybdates
Source: Materials (Basel). 2023 Oct 13;16(20):6675. doi: 10.3390/ma16206675 (PMC10608134; doi:10.3390/ma16206675)
Supplement: Supplementary file 1 [file materials-16-06675-s001.zip › materials-2620319-supplementary.pdf]

Supplementary Information

Section S1

Following figures include EDS results. Spectra were measured for surface and for cracks (refer to exemplary SEM picture on figure 1)

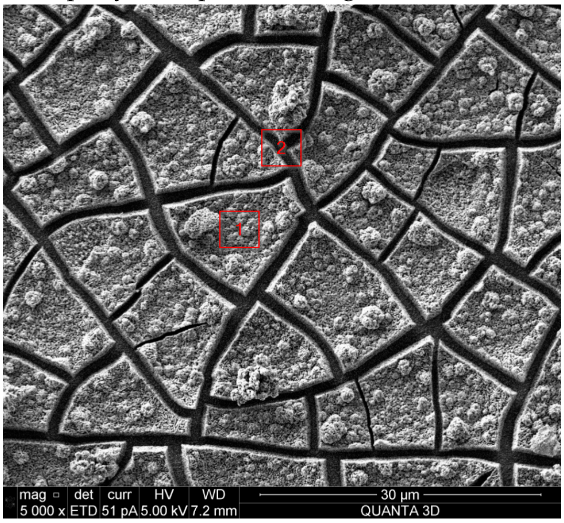

Figure S1. Exemplary SEM image depicting places for EDS analysis

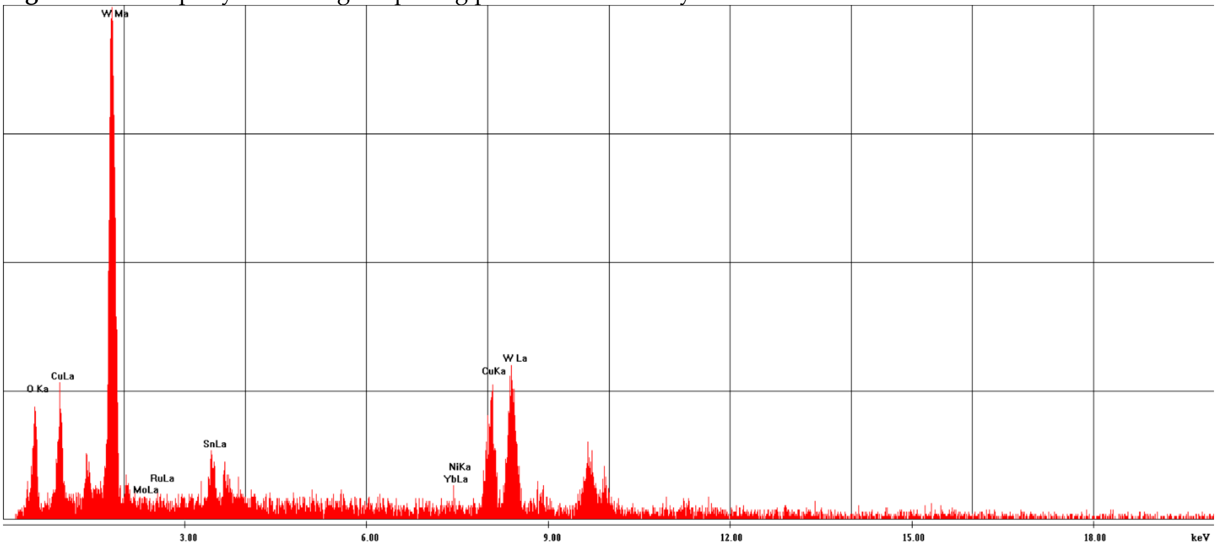

Figure S2. EDS spectrum for CW sample, place 1

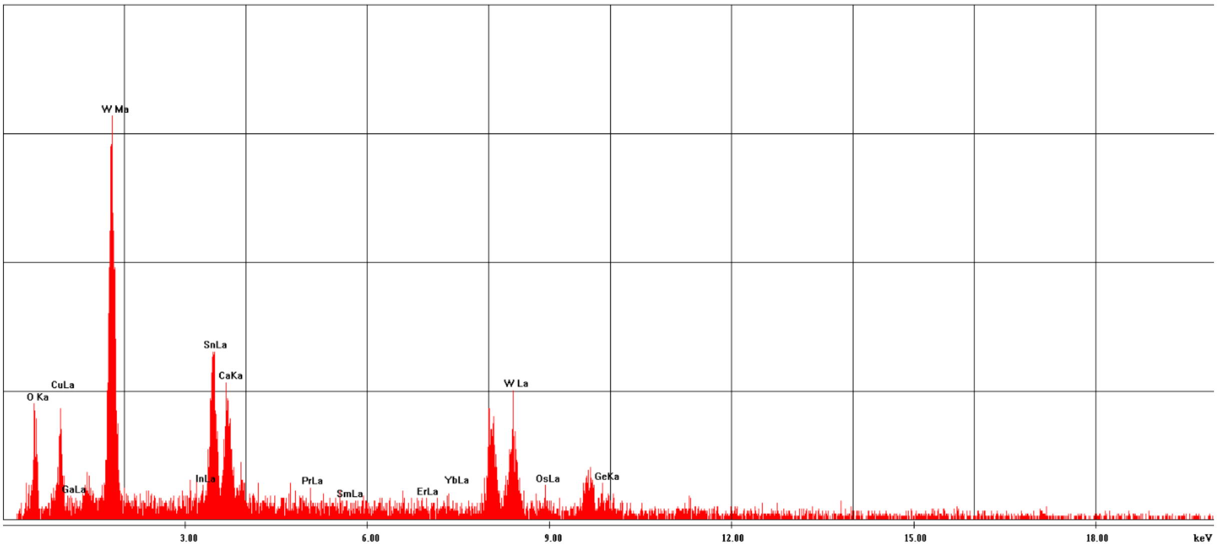

Figure S3. EDS spectrum for CW sample, place 2

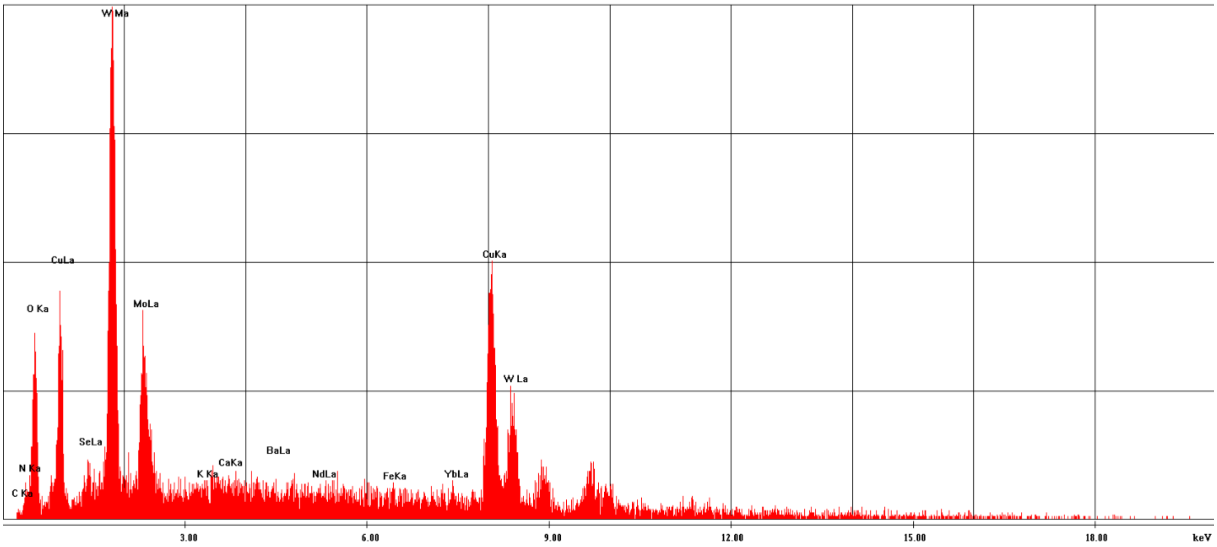

Figure S4. EDS spectrum for CWM1 sample, place 1

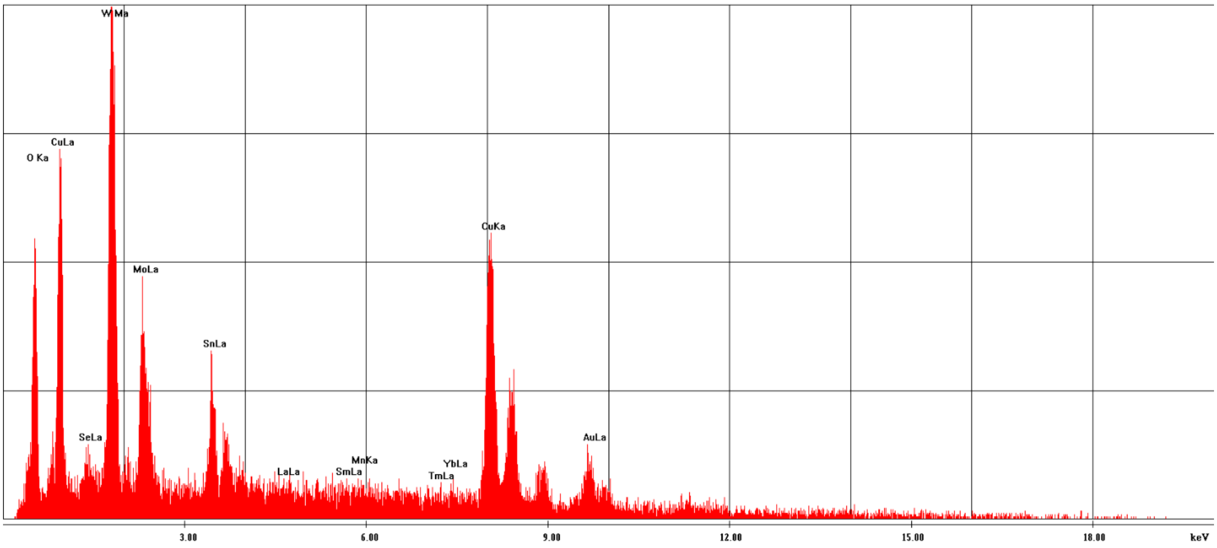

Figure S5. EDS spectrum for CWM1 sample, place 2

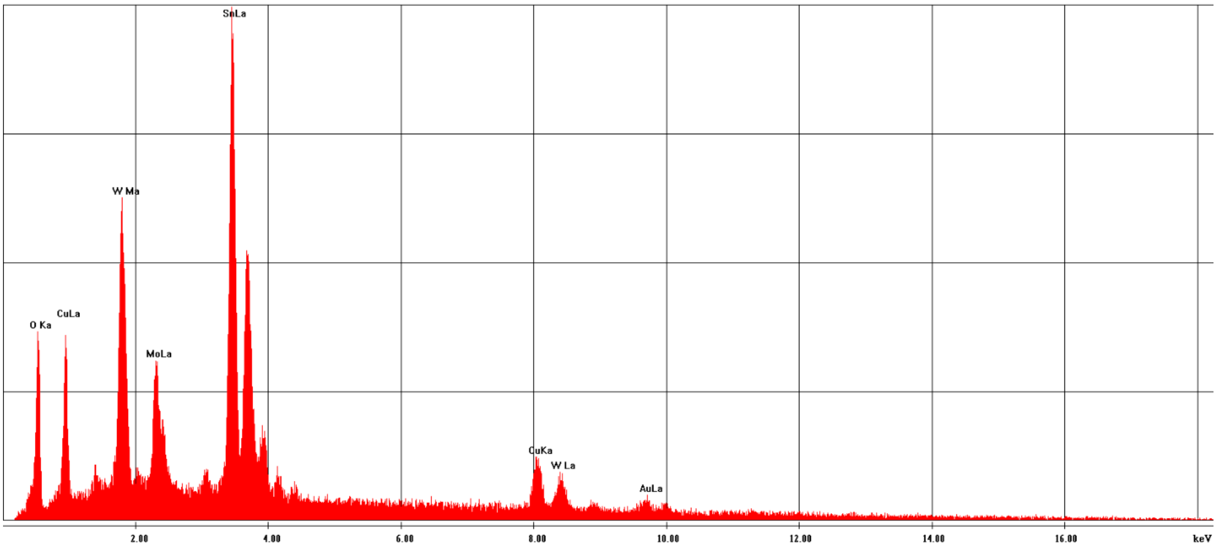

Figure S6. EDS spectrum for CWM2 sample, place 1

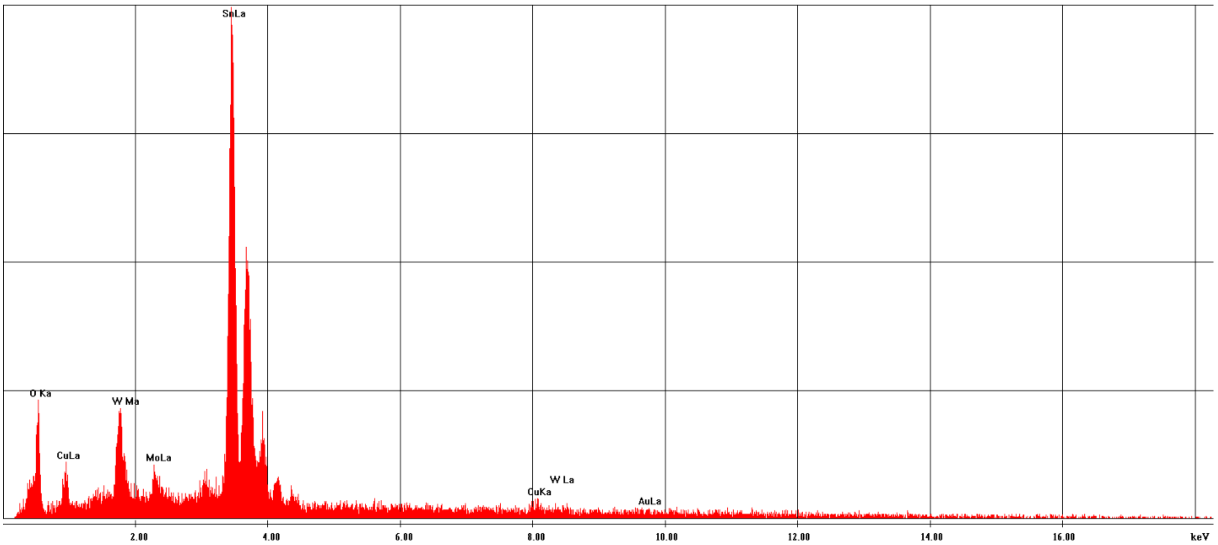

Figure S7. EDS spectrum for CWM2 sample, place 2

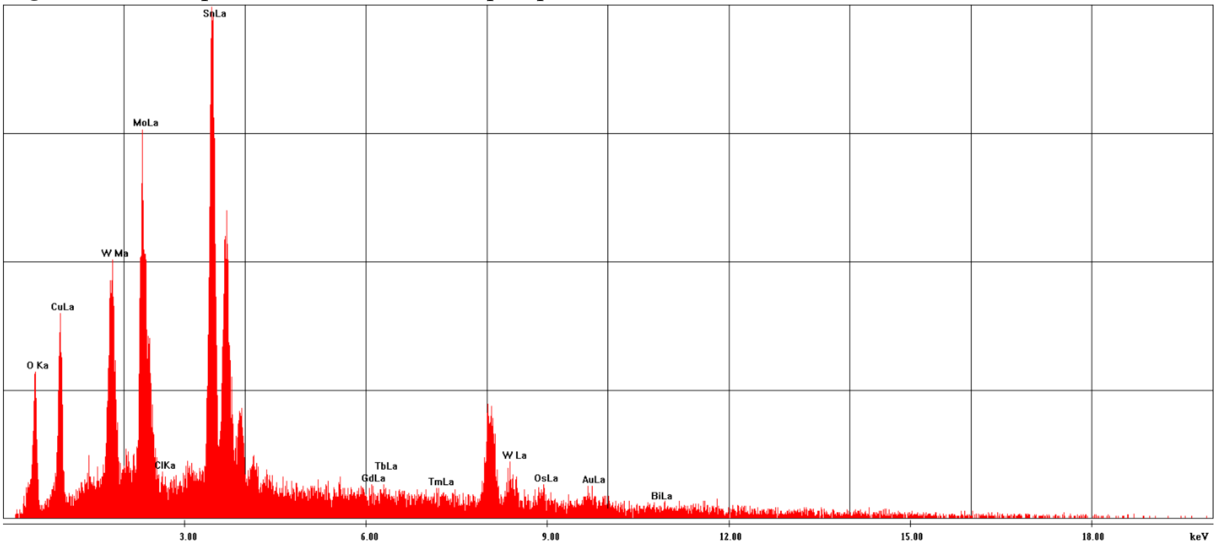

Figure S8. EDS spectrum for CWM3 sample, place 1

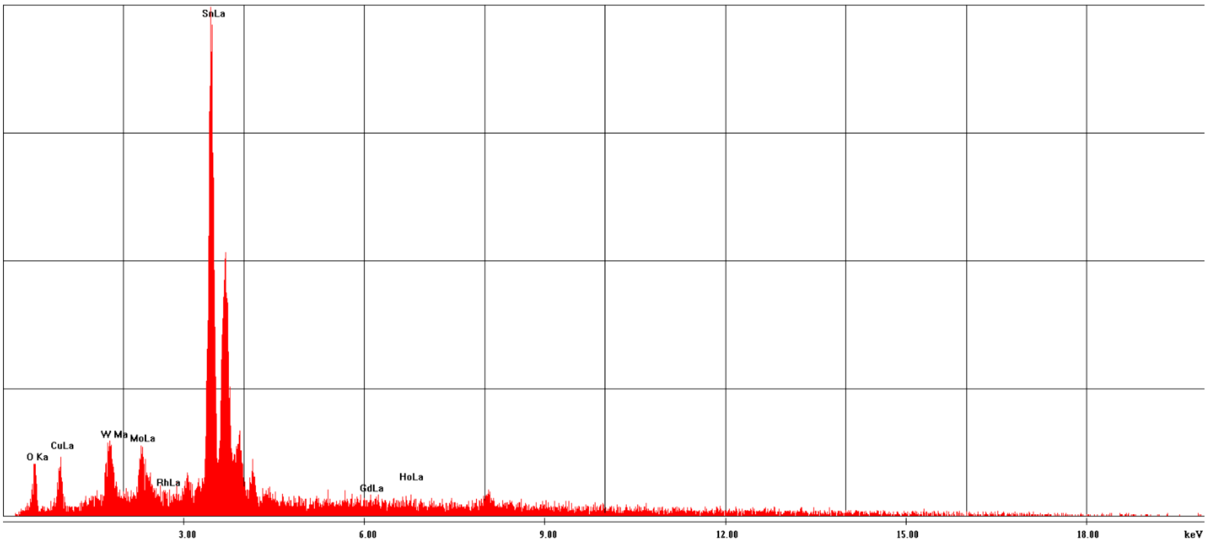

Figure S9. EDS spectrum for CWM3 sample, place 2

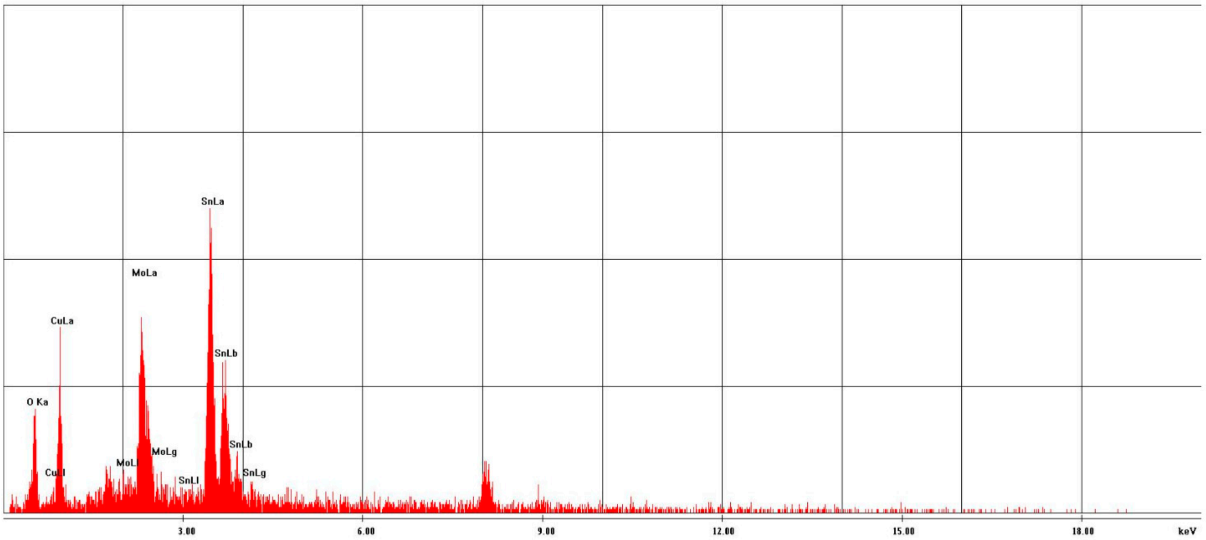

Figure S10. EDS spectrum for CM sample, place 1

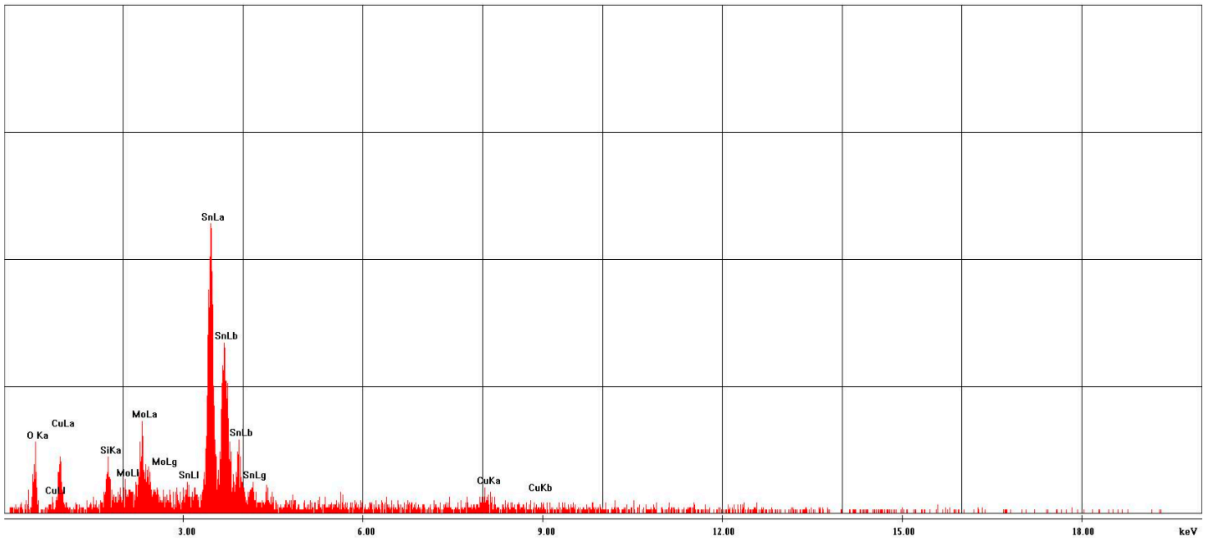

Figure S11. EDS spectrum for CM sample, place 2

Section S2

Following figures include CV measurements with different scan rate

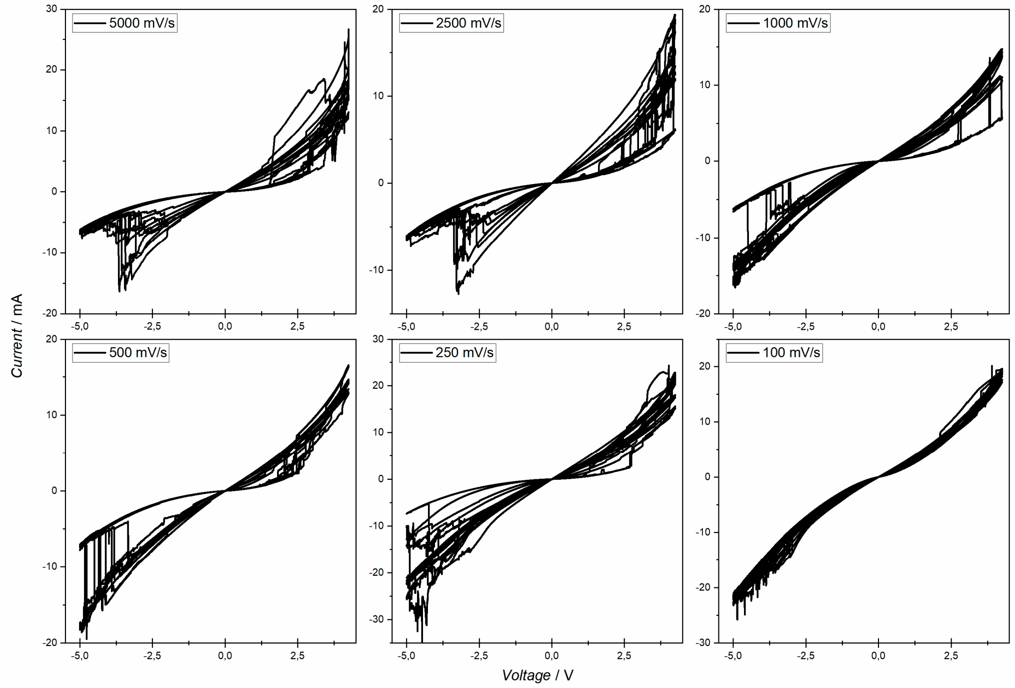

Figure S12. Cyclic Voltammetry results for CW sample

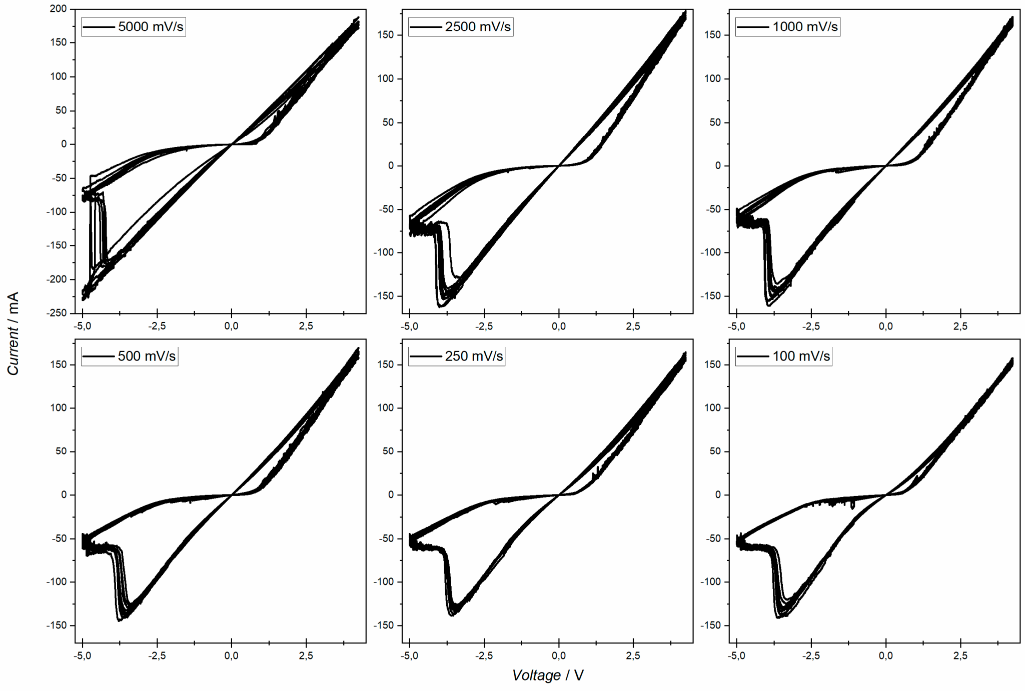

Figure S13. Cyclic Voltammetry results for CWM1 sample

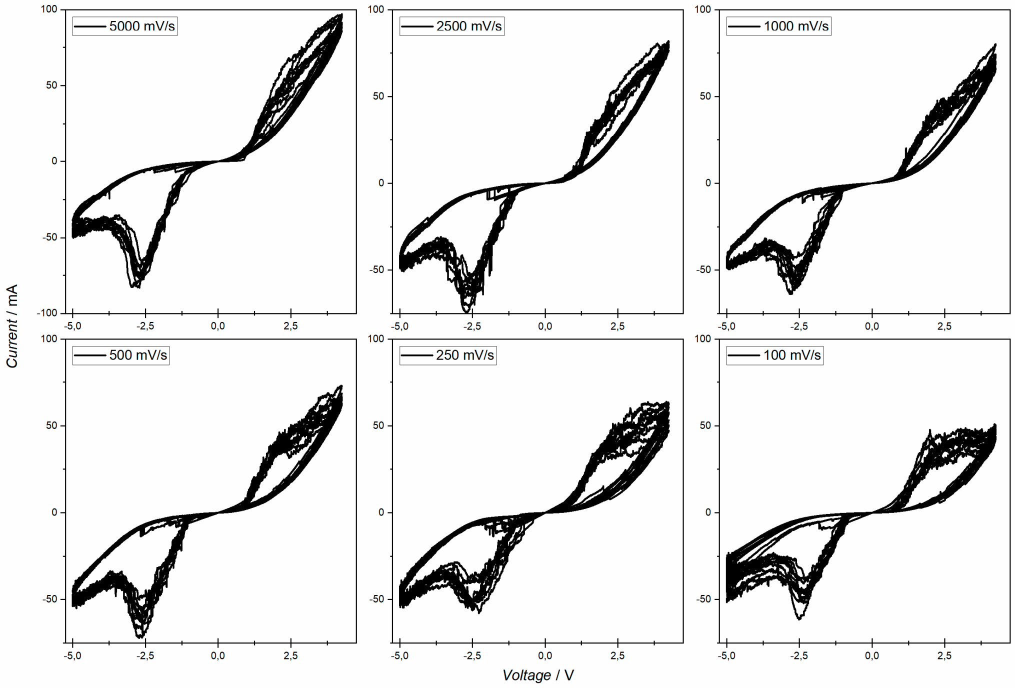

Figure S14. Cyclic Voltammetry results for CWM2 sample

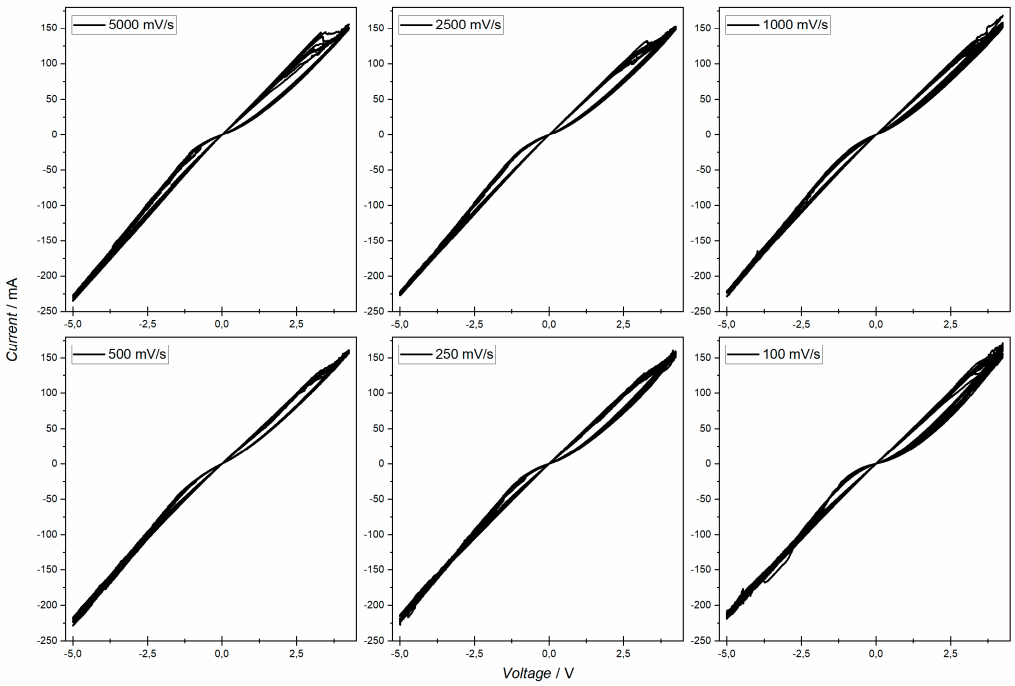

Figure S15. Cyclic Voltammetry results for CWM3 sample

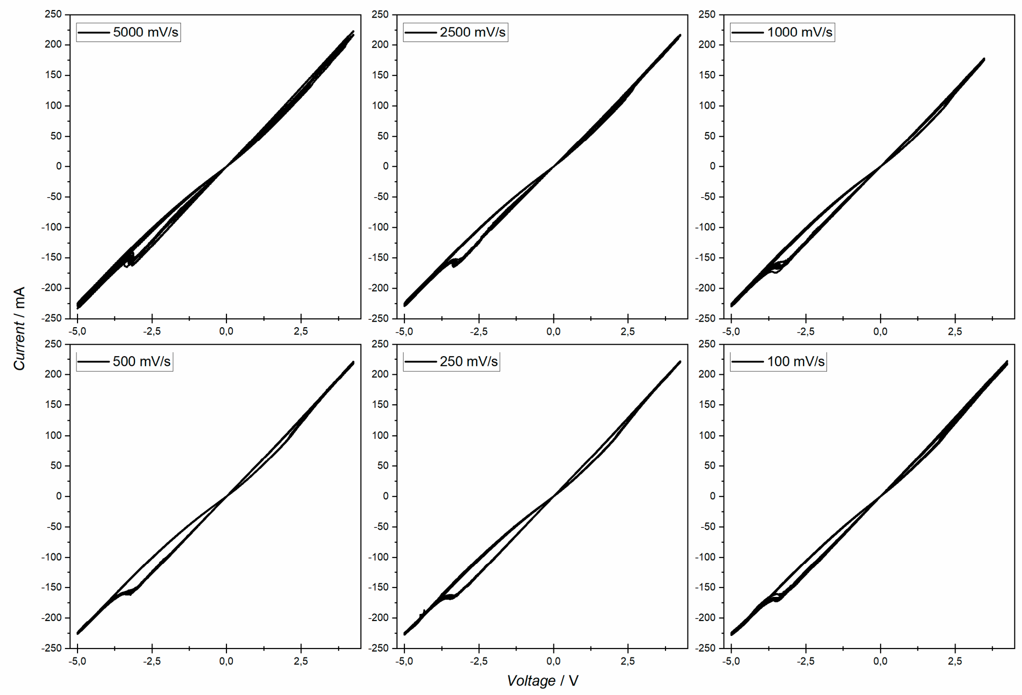

Figure S16. Cyclic Voltammetry results for CM sample

Section S3

Sequence for STDP measurements is shown on Figure 17. Each sample was measured 3 times for each Δt.

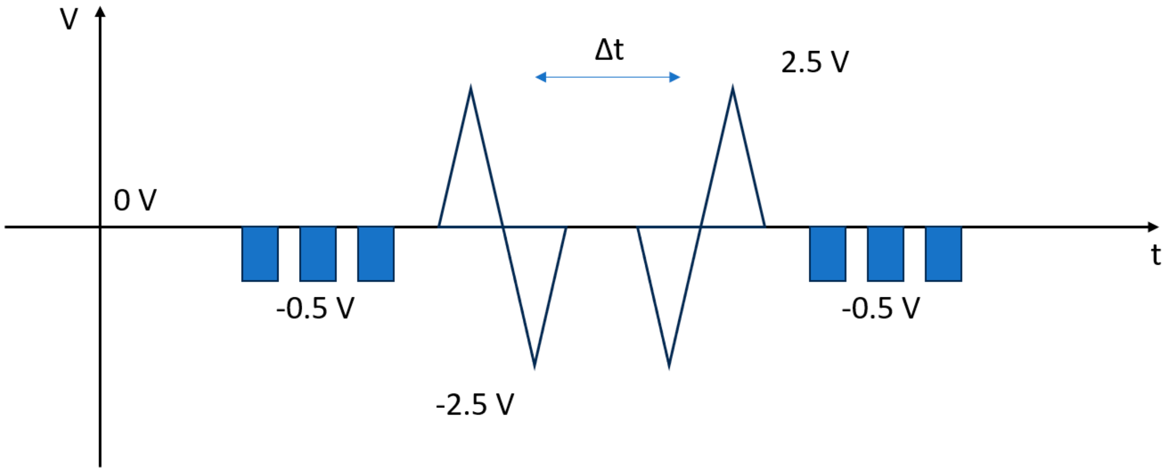

Figure S17. Voltage patterns for STDP measurements
